# Distal Xq duplication and functional Xq disomy

**DOI:** 10.1186/1750-1172-4-4

**Published:** 2009-02-20

**Authors:** Damien Sanlaville, Caroline Schluth-Bolard, Catherine Turleau

**Affiliations:** 1 Hospices Civils de Lyon, Centre de Biologie et de Pathologie Est, Service de Cytogénétique Constitutionnelle, Lyon, France; 2 Université Claude Bernard, Faculté Lyon-Nord, Lyon, France; 3 Assistance Publique- Hôpitaux de Paris, Hôpital Necker-Enfants Malades, Service d'Histo-Embryo-Cytogénétique, Université Paris Descartes, Paris, France

## Abstract

Distal Xq duplications refer to chromosomal disorders resulting from involvement of the long arm of the X chromosome (Xq). Clinical manifestations widely vary depending on the gender of the patient and on the gene content of the duplicated segment. Prevalence of Xq duplications remains unknown. About 40 cases of Xq28 functional disomy due to cytogenetically visible rearrangements, and about 50 cases of cryptic duplications encompassing the *MECP2 *gene have been reported. The most frequently reported distal duplications involve the Xq28 segment and yield a recognisable phenotype including distinctive facial features (premature closure of the fontanels or ridged metopic suture, broad face with full cheeks, epicanthal folds, large ears, small and open mouth, ear anomalies, pointed nose, abnormal palate and facial hypotonia), major axial hypotonia, severe developmental delay, severe feeding difficulties, abnormal genitalia and proneness to infections. Xq duplications may be caused either by an intrachromosomal duplication or an unbalanced X/Y or X/autosome translocation. In XY males, structural X disomy always results in functional disomy. In females, failure of X chromosome dosage compensation could result from a variety of mechanisms, including an unfavourable pattern of inactivation, a breakpoint separating an X segment from the X-inactivation centre in cis, or a small ring chromosome. The *MECP2 *gene in Xq28 is the most important dosage-sensitive gene responsible for the abnormal phenotype in duplications of distal Xq. Diagnosis is based on clinical features and is confirmed by CGH array techniques. Differential diagnoses include Prader-Willi syndrome and Alpha thalassaemia-mental retardation, X linked (ATR-X). The recurrence risk is significant if a structural rearrangement is present in one of the parent, the most frequent situation being that of an intrachromosomal duplication inherited from the mother. Prenatal diagnosis is performed by cytogenetic testing including FISH and/or DNA quantification methods. Management is multi-specialist and only symptomatic, with special attention to prevention of malnutrition and recurrent infections. Educational and rehabilitation support should be offered to all patients.

**Disease name:**

Xq duplications, Xq functional disomy

## Background – epidemiology

Duplications of the long arm of chromosome X (Xq) include intrachromosomal duplications and partial disomies/trisomies resulting from unbalanced translocations with an autosome or with a chromosome Y. As for other X-linked disorders, X-inactivation plays a major role in clinical expression of these chromosomal imbalances with usually milder symptoms in females than in males. Large, cytogenetically visible duplications of Xq are rare [1]. They implicate more often the distal Xq27-qter region [2-5]. Recently, CGH array techniques allowed the detection of smaller imbalances. Duplications including the *MECP2 *gene in Xq28 are of major concern, with about 47 cases reported in the literature. Now it seems clear that the *MECP2 *gene is the most important dosage-sensitive gene responsible for neurologic impairment in patients with duplications of the distal part of chromosome Xq. After a short reminder of X-inactivation mechanism, and general considerations about Xq duplications, we will focus mainly on Xq distal duplications.

Prevalence of Xq duplications is presently unknown. About 40 cases of Xq functional disomy due to cytogenetically visible rearrangements, and about 50 cases of cryptic duplications encompassing the *MECP2 *gene have been reported.

### Functional disomy: definition and role of X inactivation (Figures 1 and 2)

**Figure 1:**
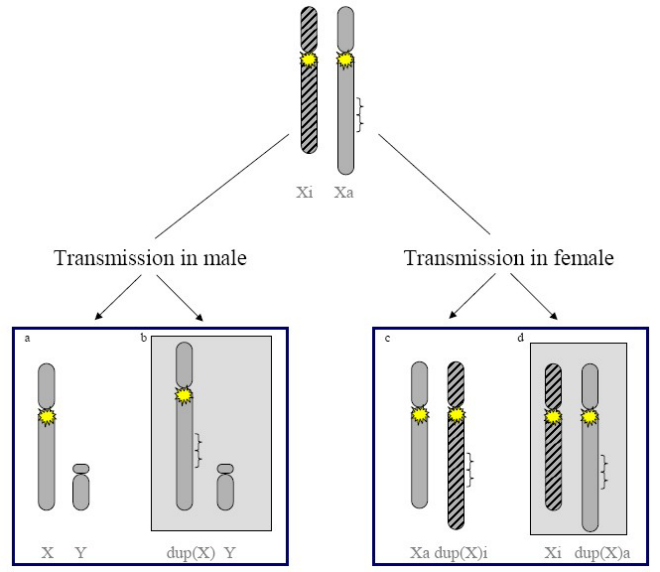
**Schematic representation of a Xq duplication transmission**. Active X and Y chromosomes are in grey, inactive X is striped in black. A yellow star represents XIC. a) normal XY chromosomes; b) Xq duplication in male leading to Xq functional disomy, associated with an abnormal phenotype; c) Xq duplication in female with inactivation pattern skewed towards the duplicated X, associated with a normal phenotype; d) Xq duplication in female with inactivation pattern skewed towards the normal X leading to Xq functional disomy, associated with an abnormal phenotype.

**Figure 2:**
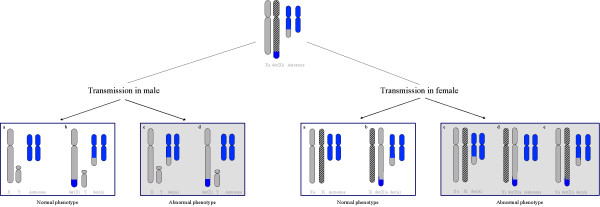
**Schematic representation of a t(X;A) transmission**. Autosomes are blue, active X chromosome and Y chromosome are grey, inactive X chromosome is striped in black. Top: balanced t(X;A) in the mother, with usual pattern of inactivation (normal X inactivated). Transmission in male: a) normal; b) balanced translocation; c) unbalanced translocation with derivative autosome, leading to functional X disomy; d) unbalanced translocation with derivative X, leading to partial X monosomy and partial autosomal trisomy; a and b are associated to a normal phenotype, c and d to an abnormal phenotype. Transmission in female: a) normal; b) balanced translocation with normal X inactivated; c) unbalanced translocation with derivative autosome, leading to partial autosomal monosomy and to functional X disomy; d) unbalanced translocation with derivative X leading to partial X monosomy and partial autosomal trisomy; e) balanced translocation with rare inactivation pattern (der(X) inactivated) leading to functional disomy and potential inactivation spreading to translocated autosomal genes; a and b are associated to a normal phenotype; c, d and e to an abnormal phenotype.

X chromosome inactivation (XCI) is the epigenetic mechanism through which mammalian cells achieve gene dosage compensation between male (XY) and female cells (XX). Most X-linked genes are therefore expressed in a similar level in females as in males. Functional disomy (FD) is the double expression of X-linked genes compared to their normal level. In males, all structural duplications lead to FD. In females, the X structural imbalances may lead to a variable phenotype depending on the X-inactivation pattern.

X-inactivation of one X chromosome occurs early in female development and is initiated from the X-Inactivation Centre (XIC), located in Xq13 on the proximal part of the long arm, and that contains the *XIST *gene. As the choice of the inactive X is a random process, each female is a functional mosaic for two cell populations [6]. The number of cells that have inactivated the same X chromosome follows a normal distribution around a mean of 50:50. In most studies, skewing and extreme skewing are arbitrarily defined as greater than or equal to 75%, and greater than or equal to 90% of cells expressing the same X chromosome, respectively. The later value is usually retained as significant for morbid manifestations. Structural abnormalities of the X chromosome such as large deletions, duplications, and unbalanced X/autosome translocations result in skewed patterns of inactivation, with the abnormal X chromosome being inactive in most or all cells [7]. Conversely, in about 95% of balanced t(X;A), the normal X chromosome is inactivated in all cells [8]. This pattern preserves the balance of expression from the X and from the autosome (Figure 2b). In some cases, the derivative containing XIC is inactivated. Consequently, these cells are functionally disomic for a part of the X chromosome. The presence of disomic cells was highly prevalent in translocations with breakpoints at Xp22 and Xq28, even though spreading of X inactivation onto the adjacent autosomal segment was noted in most of these cases [8,9]. This pattern of inactivation is usually associated with an abnormal phenotype. The outcome of the selection process against the functional disomy X seems to be the major determinant of the clinical status in most patients with balanced X-autosome translocations [10].

It is noteworthy that more than 15% of genes on the human X chromosome, including many without functional equivalent on the Y, escape from XCI. They are mainly located in the pseudo-autosomal regions at each end of the X chromosome but a small number of them are found outside these regions [11].

### X-linked disease genes

A large number of disease conditions have been associated with the X chromosome because the phenotypic consequences of a recessive mutation are revealed directly in the hemizygous male. The X chromosome contains approximately 1,100 genes and is associated with over 300 Mendelian conditions. Both the X and Y chromosomes have a remarkable enrichment of genes involved in gonadogenesis and gametogenesis. X-linked mental retardation (XLMR) is a common cause of inherited intellectual disability with an estimated prevalence of about 1/1,000 males [12]. In a recent review, Chiurazzi *et al. *listed 215 XLMR conditions including 149 with specific clinical findings of which in 82 the causal MRX gene has been identified. Among these genes, 47 were localised on the long arm [13]. Besides the *FMR1 *gene responsible for the fragile X mental retardation syndrome, the chromosomal region Xq27.3-qter harbours several genes that have been shown to be responsible for syndromic and non-syndromic forms of XLMR (*e.g. IDS, ABCD1, L1CAM, MECP2, FLNA, IKBKG, DKC,1 FMR2, GDI1, SLC6A8*, and *MECP2*) [14]. This region is at high risk for genomic instability and several of these genes are implicated in genomic disorders due to nonallelic homologous recombination between low copy repeats [15].

Consequences of over-expression of X-linked genes is not well known, with the exception of the *PLP1 *gene (whose duplication is responsible for the Pelizaeus-Merzbacher syndrome (OMIM 312080)), and, more recently, the *MECP2 *gene. *MECP2 *selectively binds to methylated DNA and mutations in the *MECP2 *gene cause the autism-spectrum neurodevelopmental disorder Rett syndrome (OMIM 312750). Its primary role was thought to form a link between DNA methylation, histone acetylation and co-repressor molecules. Evidence is now accumulating for a more expanded role of MECP2 as a multifunctional protein involved in the modulation of chromatin structure by different mechanisms. The *MECP2 *gene is expressed in many tissues, but expression is highest in the brain. During development, MECP2 expression is very low or absent in immature neurons, then it increases during neuronal maturation and is highest in post-mitotic post migratory neurons. Mild over-expression of wild-type MECP2 protein induced neurodevelopmental abnormalities in transgenic mice, and duplication of the *MECP2 *gene causes mental retardation in human males as discussed below.

## Clinical description

Clinical manifestations vary depending on the gender and on the gene content of the duplicated segment.

### Clinical manifestations in males

Distal Xq duplications are the more frequently reported. In particular, the Xq26–q28 chromosome region yields a recognisable phenotype including distinctive facial features, major axial hypotonia, severe developmental delay, severe feeding difficulties, abnormal genitalia and proneness to infections [2,4,5,16-18]. In details, these patients present:

- Growth: prenatal and postnatal growth retardation, microcephaly

- Dysmorphism: premature closure of the fontanels or ridged metopic suture, brad face with full cheeks, epicanthal folds, large ears, small and open mouth, ear anomalies, pointed nose, abnormal palate and facial hypotonia are the most common facial features

- Psychomotor retardation: severe mental retardation, absence or severely retarded speech

- Major axial hypotonia

- Spasticity

- Malformations: genitalia malformations including hypoplasic genitalia, hypospadias and/or cryptorchidism are the more frequent malformations

- Abnormal fingers and toes have been reported

- Others frequent clinical manifestations: severe feeding difficulties with gastro oesophageal reflux, excessive drooling, seizures, recurrent infections.

Proximal Xq duplications are more rarely reported. They represent an heterogeneous group of patients with variable breakpoints, most of them studied only by banding karyotyping, with some recent exceptions [1,19]. Patients show craniofacial dysmorphism, brain and neurologic abnormalities such as aberrant brain myelination, hypotonia, mental, psychomotor and growth retardation, feeding issues, hypoplasic genitalia.

Table 1 presents clinical findings found in three groups of patients, namely Xq12q24 duplications, Xq26-qter duplications (or functional disomy), and *MECP2 *interstitial microduplications.

**Table 1:** Comparison of clinical symptoms observed in three groups of patients with Xq duplications.

		Xq21q24 DF*	Xq26.3qter DF**	MECP2 duplication ***
**Number of cases**		12	21	47

**Caesarean section**		0/1	8/14	nr

**Growth**				

	Growth retardation	9/10	17/19	2/3
	Microcephaly	4/5	19/19	5/39

**Facial dysmorphism**				

	Prominent metopic suture	nr	5/15	nr
	Epicanthus fold	nr	5/6	1/8
	Large ears	nr	9/12	4/20
	Small mouth	nr	11/13	6/20
	Abnormal palate/maxillar alveolus	nr	13/14	nr
	Facial hypotonia	nr	3/4	19/28

**Neurologic outcome**				

	Hypotonia	11/11	19/19	29/32
	Developmental delay	12/12	19/19	47/47
	Absent or delayed speech	nr	11/14	46/47
	Never walked or limited walking	nr	12/14	21/34
	Spacticity	nr	3/4	17/21
	Seizures	nr	6/16	22/42

**Malformations**				

	Hypoplastic genitalia/cryptorchidism	11/11	15/19	5/10

**Others**				

	Severe feeding problems	9/9	10/14	15/29
	Gastroesophageal reflux	nr	4/7	13/17
	Constipation	nr	5/5	nr
	Small feet	nr	8/8	nr
	Digital abnormalities	5/5	13/19	6/20
	Recurrent infections	2/2	15/17	33/40

nr : not recorder

* modified from Cheng SF *et al.*[1] and Gabbett *et al.*[19]

** modified from Sanlaville *et al.*[5] and Smyk *et al.*[20]

*** modified from Ariani *et al.*[27], Meins *et al.*[28], Van Esch *et al*[26]., Freiz *et al.*[29], del Gaudio *et al.*[30], Madrigal *et al.*[31], and Smyk *et al.*[20].

Several clinical symptoms are common to the three groups, despite different gene content. However, these clinical findings are often non-specific, such as hypotonia, psychomotor delay, feeding problems and genital hypoplasia. Recurrent respiratory infections, especially recurrent pneumonia, help to distinguish Xq28 functional disomy (including *MECP2 *duplication) from other XLMR-hypotonia syndromes. The recurrent infections might result from the increased dosage of the *IRAK1 *or *IKBKG *genes generally present in the duplicated region [20].

### Clinical manifestations in female

Most Xq duplications observed in males are inherited from a mother with normal or near normal phenotype. Less frequently, Xq duplications may be found by karyotyping in manifesting females studied because of mental retardation. The most frequent manifestations found in these patients are short stature, developmental delay, facial dysmorphism and gonadal dysgenesis [21,22]. Body asymmetry may be present corresponding to functional mosaicism. In the manifesting females, the X-inactivation pattern is usually at random and this explains the clinical manifestations. In rare cases, a favourable skewed X-inactivation is observed. For these cases, other explanations such as local escape from inactivation, expression of recessive genes from the active X, or disruption of a gene by the rearrangement have been suggested to explain the abnormal phenotype [23,24]. Also a critical region for gonadal dysgenesis has been suggested to be present in Xq13q26 [25]. For duplications resulting from unbalanced t(X;A), the X-translocated segment separated from its corresponding XIC cannot be inactivated, and abnormal phenotype is fully expressed. In this rare situation, a phenotype as severe as in males is observed in female patients, with microcephaly, seizures and severe mental retardation [5].

### Specific phenotypes

Small duplications detected using array comparative genomic hybridization (array CGH) and encompassing only one or a few genes allow description of some more specific phenotypes due to over-expression of individual genes. The most striking example is the *MECP2 *gene which prove to be the major gene implicated in Xq26-qter duplications.

### *MECP2 *duplication

Recently, array CGH and quantitative polymerase chain reaction (PCR) allowed detection of submicroscopic Xq28 interstitial duplications. These microduplications are variable in size, ranging from 0.2 to 2.2 Mb, but consistently include *MECP2 *and L1 cell adhesion molecule (*L1CAM*), as well as intervening genes [26]. To date, 47 affected individuals from 24 different families have been reported [20,26-31]. The patients manifested severe mental retardation, absent or limited speech, progressive neurologic problems (such as spasticity and seizures), axial and facial hypotonia, mild and non-specific facial dysmorphism, and severe recurrent respiratory infection overlapping with characteristics described in patients with larger Xq27–q28 terminal duplications [26,30].

Collective data of previous studies suggest that increased *MECP2 *gene copy number is mainly responsible for the neurodevelopmental phenotypes in these patients [20,26,29,30]. Moreover, mice with over-expression of *mecp2 *have severe motor dysfunction [32] and progressive neurologic decline [33]. In addition, Del Gaudio *et al. *found *MECP2 *triplication in one patient with a more severe phenotype [30]. Additionally, a non-pathogenic duplication of Xq28 that does not include the *MECP2 *gene has recently been reported [34].

### SRY (sex determining region Y)-box 3 (*SOX3*) duplication

In 2005, Stankiewicz *et al. *reported a family in which five females presented with short stature, speech and language problems, hearing impairment, and several dysmorphic features associated with a 7.5-Mb duplication of Xq26.2–q27.1 that encompassed or disrupted the *SOX3 *gene [35]. *SOX3 *gene has previously been suggested as a candidate gene in a female with moderate to severe mental retardation, seizures and hypothyroidism and del(X)(q26.3–q27.3) [36]. At the same time, Woods *et al. *identified a 685 kb submicroscopic duplication of Xq27.1 containing the *SOX3 *gene in two maternal half males sibs with X-linked panhypopituitarism [37]. Brain magnetic resonance imaging (MRI) showed anterior pituitary hypoplasia, ectopic posterior pituitary, and absent infundibulum. They concluded that both over- and underdosage of *SOX3 *are associated with similar phenotypes, consisting of infundibular hypoplasia and hypopituitarism but not necessarily mental retardation.

### Oligophrenin 1 (*OPHN1*) duplication

Recently, using oligonucleotide array CGH, Bedeschi *et al. *reported a familial chromosome duplication spanning about 800 kb of genomic DNA encompassing the *YIPF6*, *STARD8 *and *OPHN1 *genes. The male proband had microcephaly, a distinct facial appearance, severe mental retardation and language impairment [38]. *OPHN1 *mutations have been reported in patients with moderate to severe mental retardation, myoclonic-astatic epilepsy, ataxia, cerebral hypoplasia strabismus and hypogenitalism [39]. The observation of Bedeschi *et al., *stressed the interest of high resolution array CGH to delineate new clinical phenotype secondary to small chromosome abnormalities, in particular duplication.

### Proteolipid protein 1 (*PLP1*) duplication

Microduplications encompassing the *PLP1 *gene have also been reported with a clinical phenotype evocative of Pelizaeus-Merzbacher disease (PMD) [40]. Pelizaeus-Merzbacher disease (OMIM 312080) is a very rare X-linked recessive inherited leukodystrophy with prevalence estimated at 1 out of 400,000 birth. The disease occurs as an early motor development impairment marked by hypotonia associated with nystagmus, ataxochoreic movements of the axis and limbs, especially during the first 2 years of life. Symptoms often progress slowly until adolescence. PMD is caused by mutation in the gene encoding the proteolipid protein-1 (*PLP1*) gene. Complete duplication of the *PLP1 *gene on Xq22 is the cause of 60–70% of PMD cases, whereas deletions of this gene as well as point mutations in coding or splice site regions are involved in most of the remaining cases [41].

### Other genes duplications

In addition, Froyen *et al.*, using array CGH, reported two other small Xq duplications. The first one is a duplication of about 0,8 Mb at Xq22 containing 15 annotated genes. They proposed the *NXF5 *gene, including in the duplicated region, to explain mental retardation. In fact, increased dosage of NXF5 might disturb storage levels of particular mRNA in neurons. Nevertheless, expression levels of NXF5 mRNA in the patient compared to controls could not be investigated. The second, is a small 0,3 Mb duplication harbouring 12 known genes including the XLMR genes *FLNA *and *GDI1 *suggesting a dosage-dependant role for at least one of both genes [42].

## Aetiology

In XY males, structural X disomy always results in functional disomy and may be caused either by an intrachromosomal duplication or an unbalanced X/Y or X/autosome translocation. In females, failure of X chromosome dosage compensation could result from a variety of mechanisms, including an unfavourable pattern of inactivation, a breakpoint separating an X segment from the X-inactivation centre in cis, or a small ring chromosome.

### Intrachromosomal duplications (Figure 1)

Intrachromosomal duplications are the main cause of functional disomy in males. In most cases, they are inherited and transmitted in families through non-manifesting mothers. They are very variable in size, location and, therefore, in gene content. Before the use of array CGH, only cytogenetically visible duplications of at least 5–10 Mb in size were diagnosed. Duplications encompassing the X-inactivation centre are subject to inactivation, as in the rare variant of Klinefelter syndrome with isochromosome Xq [43]. An unusual type of intrachromosomal duplication with the Xq26.3 or Xq27 ->qter region translocated to the Xp22.3 band is observed in some cases. It is usually inherited or may derive from a recombination within a maternal pericentric inversion. CGH array allowed detection of far more smaller duplications that may encompass only one or few genes, or even be asymptomatic [42].

In females, intrachromosomal duplications of X chromosome are generally associated with a skewed inactivation pattern biased towards the duplicated X chromosome leading to a normal or near normal phenotype (Figure 1c), and they are detected through abnormal offspring. However, rare cases with a random X inactivation ratio have also been described in female patients with mental retardation/multiple congenital anomalies (MR/MCA) syndromes [44].

Recombination between genomic repeats is a possible mechanism for the origin of the non-recurrent duplications. Giglio *et al. *described four der(X) chromosomes and proposed recombination between inverted repeat sequences during paternal meiosis as mechanism of origin [45]. However, non-recurrent aberrations can result from different non-exclusive recombination-repair mechanisms. The origin and mechanisms underlying some of the Xq duplications have been investigated in cloning their breakpoints. Bauters *et al. *have analysed the breakpoints of 16 unique microduplications containing *MECP2 *and shown that none of these duplications are the result of NAHR. They demonstrated non-homologous end joining (NHEJ) as the mechanism in one of them, and a complex two-step rearrangement involving breakage-induced replication with strand invasion of the normal chromatid in another one [46]. In Pelizaeus-Merzbacher disease, complex non-recurrent rearrangements are observed that are likely to be caused by a replication mechanism involving template switching [47].

### Translocations (Figure 2)

Duplications resulting from an unbalanced form of X-Y [t(X;Y)] or X-autosome translocations [t(X;A)] are more rarely reported, with imbalance limited to the distal portion of Xq.

About six cases of t(X;Y) have been reported in severely retarded males with non-fluorescent Y chromosome and translocation of about 10 Mb of Xqter onto Yq. All cases occurred *de novo *[5,20,42].

X-autosome translocations [t(X;A)] are rare rearrangements estimated to occur in 1 to 3/10,000 live births [8,9]. Xq duplications resulting from unbalanced t(X;A) have been observed in males and in females for small terminal Xq segments translocated onto an acrocentric short arm, or very distally onto another chromosome arm. In this type of t(X;A), the autosomal imbalance is absent or very limited, and does not impact on the phenotype. In females, the X chromosome segment, separated from the X-inactivation centre in cis, cannot be inactivated and this result in functional disomy whatever the inactivated X. Theoretically, some balanced t(X;A) may result in Xq functional disomy if the derivative X is inactivated. In any case, parental karyotypes are required to eliminate an inherited translocation.

### Small X ring chromosomes

Small X ring chromosomes [r(X)] lacking functional XIC fail to be inactivated. Depending on their gene content, they may be responsible for FD resulting in a more severe phenotype than Turner syndrome, with severe mental retardation [48,49].

## Diagnosis and diagnostic methods

### Karyotype

The patients are usually investigated for mental retardation and a karyotype is performed using banding techniques that detect cytogenetically visible structural chromosome rearrangement such as large duplications, or X ring chromosomes. In most cases, the karyotype resolution is not sufficient, and Xq duplication will be detected applying additional techniques.

### DNA quantitative techniques

DNA techniques such as quantitative PCR (qPCR), multiplex ligation-dependent probe amplification (MLPA) or QM-PSF (quantitative multiplex PCR of short fluorescent) can be used to detect a targeted Xq duplication, in particular for the *MECP2 *or the *PLP1 *locus.

### FISH

FISH (Fluorescence *in situ *hybridisation) can be used after an initial diagnosis done by karyotype, and selection of a specific probe. Nevertheless, diagnosis of duplication using FISH could be difficult, in particular in prenatal setting. Therefore, quantitative methods are often much better ways of detecting small duplications.

### Array CGH

Today, CGH array is rapidly becoming the method of choice to detect small genomic imbalances [50]. This technique can detect small duplications (few kb) as well as large duplications (several Mb) allowing description of more specific phenotypes such as *MECP2 *duplication phenotype.

Since 30% more males than females are diagnosed with mental retardation, full coverage X chromosome CGH-arrays have been developed for the detection of copy number alterations in patients with suspected X-linked mental retardation (XLMR) [51]. Froyen *at al. *recently reported a cohort of 108 patients screened using a tiling X-chromosome-specific genomic array with a theoretical resolution of 80 kb and detected a total of 15 copy number changes in 14 patients (13%) that included two deletions and 13 duplications ranging from 0.1 to 2.7 Mb harbouring the *CDKL5, NXF5, MECP2*, and *GDI1 *genes [42]. Also, Madrigal *et al. *detected 26 copy number variants (CNVs) on 52 patients using X-chromosome tiling path array [31]. For 8 patients (14,8%) they identified imbalances probably causative of the phenotype observed in the patients. In particular, they reported one patient with Xq12 deletion, one patient with Xq12 duplication and two patients with Xq28 duplication. The genomic size of chromosome rearrangement ranged between 82 to 700 kb.

Whole genome CGH arrays with variable resolution are more generally used to study patients with unexplained MR/MCA and may lead to an increasing number of Xq duplication diagnosis.

### Molecular X-inactivation studies

X-inactivation studies are required in carrier females. In the prenatal period and in infants, it may help to the prognosis. For small duplications of uncertain significance found by CGH array, a random inactivation in an asymptomatic carrier mother argues against a pathogenic effect of this duplication. X-inactivation studies are most often done using a PCR protocol based on the methylation status of X chromosomes [52]. Alternatively, late replication assay by cytogenetic methods using BrdU incorporation distinguishes the two X chromosomes according to their morphology (normal or rearranged).

## Differential diagnosis

Among syndromes presenting clinical features in common with Xq duplications we will discuss the following:

### Prader-Willi syndrome (PWS)

Prader-Willi syndrome (OMIM 176270) affects 1 out of 25,000 births. It is characterised by two periods: I) neonatal period with severe hypotonia, characteristic facial features, feeding difficulties, hypogenitalism, and small hands and feet; ii) infancy and adulthood period: hyperphagia with a risk of morbid obesity, learning difficulties and behavioural problems. The expert consensus is that diagnosis should be based on clinical criteria (Holm's criteria of 1993, revised in 2001 [53]). PWS is caused by anomalies involving the critical region of chromosome 15 (15q11–q13). The syndrome results from absence of paternally active gene expression at the 15q11q13 chromosomal region [53]. Phenotypic features of PWS have previously been reported in association with distal Xq duplication [2,17,54] and interstitial Xq duplication [19]. It has been suggested that proximal Xq duplications can result in features typically seen in children and adults with PWS (obesity and hypogonadism), and duplications distal to Xq25 to the infantile features of PWS in males (severe hypotonia, feeding difficulties and hypogenitalism) [19].

### Alpha-thalassaemia mental retardation

X-linked alpha thalassaemia mental retardation (ATR-X) syndrome (OMIM 301040) presents with many of the clinical features found in patients with Xq duplication: males with profound developmental delay (language is usually very limited), facial dysmorphism, genital abnormalities (in 80% of children, ranging from undescended testes to ambiguous genitalia). In this syndrome alpha thalassaemia is occasionally observed. This syndrome is X-linked recessive and results from mutations in the *ATRX *gene [55].

## Genetic counselling

In all cases, parental karyotypes are necessary prior to genetic counselling. The recurrence risk is significant if a structural rearrangement is present in one of the parent, usually the mother. Genetic counselling depends on the type of chromosomal rearrangement, on the sex of the carrier and on the mechanisms potentially leading to functional disomy. Periodic genetic evaluations are warranted to update parents as more information on this syndrome becomes available.

### Intrachromosomal duplications

The majority of Xq duplications are inherited from a heterozygous mother [56]. Most heterozygous females show extreme to complete skewing of X chromosome inactivation and thus are asymptomatic. The risk for siblings depends on the carrier status of the mother.

If the mother is a carrier, the risk of transmitting the duplication is 50% for each pregnancy (Figure 1). All XY sons will be affected, daughters will be usually asymptomatic heterozygous carriers, but may be more or less severely affected depending on their X-inactivation pattern.

If the duplication had arisen *de novo*, the recurrence risk for siblings is not significantly increased when compared with that of the general population. However, prenatal diagnosis may be counselled because cryptic or germinal mosaicism may be present in the mother.

### X;Y and X;Autosome translocations

Here again, the risk for siblings depends on the carrier status of the parents, with a majority of cases occurring *de novo*. Translocation of a small segment of Xq onto Yq results in all cases in FD and always occurs *de novo*. X;A translocations are frequently associated with infertility, but may be sometimes present in a balanced form in the mother. In this specific situation, there is a substantial risk of having abnormal offspring due to an unbalanced chromosomal constitution. In unbalanced X;A translocations, X-inactivation of the X segment translocated onto the autosome cannot occur, in females as in males (Figure 2c). The balanced form of the translocation may also be transmitted to daughters with possible detrimental effect due to an unfavourable pattern of inactivation. This requires evaluation of each individual case by a specialist.

## Prenatal testing

### Prenatal diagnosis with index case

Chromosomal rearrangement can be detected by amniocentesis or chorionic villus sampling and cytogenetic testing including FISH and/or DNA quantification as performed in the propositus. The most frequent situation is that of an intrachromosomal duplication inherited from the mother. Prenatal testing may also be done after diagnosis of a *de novo *case to exclude germinal mosaicism. For girls receiving the duplication, the phenotype depends on X-inactivation pattern. X-inactivation study in prenatal period is possible, with cautious interpretation. For pregnancies in which the mother has been identified as a carrier of an interstitial *MECP2 *duplication, the usual procedure should be used: determination of foetal sex and testing for the *MECP2 *duplication in male. For t(X;A), careful examination of individual situation is required.

### Prenatal diagnosis without index case

Large Xq duplications could be diagnosed by chance after foetal karyotyping for any reason. No antenatal ultrasound signs specific to Xq duplications have been described.

## Management

Individuals with Xq duplication should receive routine medical care from their primary physician. For symptoms affecting particular organ systems, specialists should be consulted. Management is only symptomatic. Patients are generally treated in the same manner as other patients presenting the same clinical manifestations, without Xq duplication. Nevertheless, the increase of knowledge on the syndrome will support the educational and rehabilitation aid by parents and caregivers.

The swallowing difficulties need an assessment of feeding in infancy. Early parenteral nutrition could limit postnatal growth retardation. In fact, various strategies (including parenteral nutrition) should be investigated to reduce and if possible, prevent malnutrition [5]. Speech and communication therapy is essential. Augmentative communication aids, such as picture cards or communication boards, should be used at the earliest appropriate time. Elucidation of the type and frequency of seizures is accomplished by clinical history and electroencephalography. Anticonvulsivant medication should be used as for seizures in the general population.

Major milestones should be evaluated and addressed in a standard manner, often including a physical therapy evaluation. Because of spasticity, physical therapy can help maintaining joint range of motion and prevent secondary contractures. Brain MRI could be proposed to detect cerebral malformation. In fact, grey matter heterotopia, mild Dandy-Walker variant, and corpus callosum agenesis have been reported [29]. The susceptibility of affected males with Xq28 duplication to respiratory infection raises the question regarding the use of prophylactic antibiotics. The use of antibiotics during the winter may be beneficial in patients with Xq28 duplications [29].

## Prognosis

As far as we know, the oldest patient reported so far is 30-years-old. Friez *et al. *reported that six patients with *MECP2 *duplication out of 11 (55%) died before 25 years of age [29]. Several patients died following infection in infancy. Malformations do not contribute significantly to the morbidity associated with this syndrome.

## Conclusion

Xq duplications are rare events. Xqter duplications encompassing the *MECP2 *gene are the most frequently detected. These patients present severe mental retardation, growth retardation, facial dysmorphism, genital hypoplasia and recurrent infections. A growing number of Xq duplications are now described following the widespread use of array CGH techniques. There is no doubt that new clinically recognisable syndromes will soon be described, allowing targeted diagnosis. Candidate genes will emerge and exploring gene expression in these patients will allow a better understanding of X-linked mental retardation mechanisms, including functional disomy.

## Support: associations – internet links

A list of groups that support patients, families, and clinicians caring for patients with Xq duplications is supplied in Additional file 1.

## Abbreviations

(Xq): chromosome X; (XCI): X chromosome inactivation; (FD): Functional disomy; (XIC): X-Inactivation Centre; (XLMR): X-linked mental retardation; (array CGH): array comparative genomic hybridization; (PCR): polymerase chain reaction; (MRI): magnetic resonance imaging; (MR/MCA): mental retardation/multiple congenital anomalies; (NHEJ): non-homologous end joining; (qPCR): quantitative PCR; (MLPA): multiplex ligation-dependent probe amplification; QM-PSF: (quantitative multiplex PCR of short fluorescent), (CNVs): copy number variants; (PWS): Prader-Willi syndrome; (ATR-X): X-linked alpha thalassaemia mental retardation

## Competing interests

The authors declare that they have no competing interests.

## Authors' contributions

All authors read and approved the final manuscript.

## Supplementary Material

Additional file 1 **Support: Associations – Internet links**. This file contains a list of groups that support patients, families, and clinicians caring for patients with Xq duplications. Click here for file
