# Supplementary material for: Distal Xq duplication and functional Xq disomy
Source: Orphanet J Rare Dis. 2009 Feb 20;4:4. doi: 10.1186/1750-1172-4-4 (PMC2649904; doi:10.1186/1750-1172-4-4)
Supplement: Additional file 1 — Support: Associations – Internet links. This file contains a list of groups that support patients, families, and clinicians caring for patients with Xq duplications. [file 1750-1172-4-4-S1.doc]

# Associations – Internet links

**Unique:** The Rare Chromosome Disorder Support Group

PO Box 2189, Caterham

SURREY CR3 5GN

ROYAUME-UNI

<http://www.rarechromo.org/html/home.asp>

**Valentin:** Association de Porteurs d'Anomalies Chromosomiques

52 La Butte Eglantine

95610 ERAGNY

FRANCE

<http://www.valentin-apac.org/>

**ECARUCA:** European Cytogeneticists Association Register of Unbalanced Chromosome Aberrations

www.ecaruca.net/

**Orphanet:** The portal for rare diseases and orphan drugs

<http://www.orpha.net/>

DECIPHER: https://decipher.sanger.ac.uk/

GENETEST: http://www.geneclinics.org/

MECP2 duplication: http://www.mecp2duplication.com/

GENATLAS: www.genatlas.org
